# Design of Alphavirus Virus-Like Particles Presenting Circumsporozoite Junctional Epitopes That Elicit Protection against Malaria

**DOI:** 10.3390/vaccines9030272

**Published:** 2021-03-18

**Authors:** Joseph R. Francica, Wei Shi, Gwo-Yu Chuang, Steven J. Chen, Lais Da Silva Pereira, S. Katie Farney, Barbara J. Flynn, Li Ou, Tyler Stephens, Yaroslav Tsybovsky, Lawrence T. Wang, Alexander Anderson, Zoltan Beck, Marlon Dillon, Azza H. Idris, Nicholas Hurlburt, Tracy Liu, Baoshan Zhang, Carl R. Alving, Gary R. Matyas, Marie Pancera, John R. Mascola, Peter D. Kwong, Robert A. Seder

**Affiliations:** 1 Vaccine Research Center, National Institute of Allergy and Infectious Diseases, National Institutes of Health, Bethesda, MD 20892, USA; joe.francica@nih.gov (J.R.F.); shiw@mail.nih.gov (W.S.); gwo-yu.chuang@nih.gov (G.-Y.C.); steven.chen2@nih.gov (S.J.C.); lais.dasilvapereira@nih.gov (L.D.S.P.); katie.farney@nih.gov (S.K.F.); bflynn@mail.nih.gov (B.J.F.); li.ou@nih.gov (L.O.); lawrence.wang@nih.gov (L.T.W.); dillonm@mail.nih.gov (M.D.); azza.idris@nih.gov (A.H.I.); tracy.liu@nih.gov (T.L.); baoshan.zhang@nih.gov (B.Z.); mpancera@fredhutch.org (M.P.); jmascola@nih.gov (J.R.M.); 2 Electron Microscopy Laboratory, Cancer Research Technology Program, Frederick National Laboratory for Cancer Research Sponsored by the National Cancer Institute, Frederick, MD 21701, USA; tyler.stephens@nih.gov (T.S.); yaroslav.tsybovsky@nih.gov (Y.T.); 3 Laboratory of Adjuvant & Antigen Research, US Military HIV Research Program, Walter Reed Army Institute of Research, Silver Spring, MD 20910, USA; aanderson@hivresearch.org (A.A.); zoltan.beck@gmail.com (Z.B.); calving@hivresearch.org (C.R.A.); gmatyas@hivresearch.org (G.R.M.); 4 Vaccine and Infectious Disease Division, Fred Hutchinson Cancer Research Center, Seattle, WA 98109, USA; nhurlbur@fredhutch.org

**Keywords:** junctional epitope, malaria, neutralizing antibodies, parasite, *Plasmodium falciparum*, vaccines, virus-like particle

## Abstract

The most advanced malaria vaccine, RTS,S, includes the central repeat and C-terminal domains of the *Plasmodium falciparum* circumsporozoite protein (PfCSP). We have recently isolated human antibodies that target the junctional region between the N-terminal and repeat domains that are not included in RTS,S. Due to the fact that these antibodies protect against malaria challenge in mice, their epitopes could be effective vaccine targets. Here, we developed immunogens displaying PfCSP junctional epitopes by genetic fusion to either the N-terminus or B domain loop of the E2 protein from chikungunya (CHIK) alphavirus and produced CHIK virus-like particles (CHIK-VLPs). The structural integrity of these junctional-epitope–CHIK-VLP immunogens was confirmed by negative-stain electron microscopy. Immunization of these CHIK-VLP immunogens reduced parasite liver load by up to 95% in a mouse model of malaria infection and elicited better protection than when displayed on keyhole limpet hemocyanin, a commonly used immunogenic carrier. Protection correlated with PfCSP serum titer. Of note, different junctional sequences elicited qualitatively different reactivities to overlapping PfCSP peptides. Overall, these results show that the junctional epitopes of PfCSP can induce protective responses when displayed on CHIK-VLP immunogens and provide a basis for the development of a next generation malaria vaccine to expand the breadth of anti-PfCSP immunity.

## 1. Introduction

Malaria is a global health burden that afflicts over 200 million people each year, causing an estimated 405,000 deaths in 2018 [1]. The etiological agent of malaria is the *Plasmodium* parasite, several species of which can infect humans. *Plasmodium falciparum* (*Pf*), which is found primarily throughout sub-Saharan Africa and Southeast Asia, causes the most severe human disease, with children and pregnant women being most susceptible [2]. The past decade has seen significant progress in disease control, with a combination of measures including insecticide-treated bed nets and seasonal malaria chemoprevention [3]. However, in recent years this progress has stalled and is threatened by the rise of drug resistance [4,5,6].

Given the persistence of the malaria threat, a potent and durable preventative vaccine represents the most desirable public health intervention [7]. Indeed, vaccine candidates are being evaluated clinically against various stages of the parasite life cycle, including pre-erythrocytic [8,9,10], blood [11,12], and transmission-blocking stages [13,14,15]. To date, the most successful candidates have targeted the sporozoite form of the parasite, which is deposited into the skin by mosquito bite to begin the pre-erythrocytic phase of infection. Intravenous immunization with radiation attenuated sporozoites (PfSPZ vaccine) is an approach based on inducing T cells in the liver to eliminate infected hepatocytes and has been shown to protect 92% of naïve adults in the US at 3 weeks and 65% at 24 weeks [9,16], but only 52% of Malian adults [17] and 20% of Tanzanian children following controlled human malaria infection [18]. The most advanced vaccine candidate is RTS,S, which displays the major repeating tetrapeptide “NANP” and C-terminal regions of the *Pf* circumsporozoite protein (PfCSP) on hepatitis B surface antigen particles (Mosquirix, GSK vaccines) [19], and is designed to induce antibodies to prevent infection in the liver. When administered with the AS01 adjuvant three times [20], clinical protection in 5–17 month old children was ~ 50% at one year and 36% over a period of four years [21,22].

Given the more limited durability of protection of RTS,S/AS01, there are several potential possibilities to improve efficacy. Antibody responses to PfCSP have been shown to correlate with efficacy following RTS,S [23,24,25]. The PfCSP major NANP repeats are known to induce directly neutralizing antibodies, though serum reactivity to the PfCSP C-terminus is also correlated with protection and may contribute to parasite opsonophagocytosis [26,27,28]. In terms of using the vaccine regimen to alter the quality of the antibody response, delaying and fractioning the last dose of RTS,S has been shown to improve serum avidity and protection [29,30]. Another potential approach toward improving the potency of RTS,S is to display a more dense array of the immunogen on hepatitis B surface antigen particles, which is termed R21. R21 has been shown to be highly immunogenic and is currently being evaluated clinically [8].

A different approach to improve upon the efficacy of RTS,S is to increase the breadth of epitopes against which antibody responses can be elicited. Subsequent to the development of R21, we described a new neutralizing epitope on PfCSP, termed the junctional epitope, which is targeted by the highly potent human monoclonal antibody (mAb) CIS43, derived from a subject vaccinated with the PfSPZ vaccine [31]. Other groups have subsequently cloned mAbs that react with the PfCSP junction following vaccination of Tanzanian volunteers [32] and also from immunized Kymab mice [33]. Interestingly, because this new epitope is located at the junction of the N-terminal and repeat domains, it is not present in RTS,S or R21. Furthermore, we have recently described mAb L9, which recognizes a second new epitope in PfCSP formed by the minor repeat tetrapeptide, NVDP, interspersed with NANP [34]. Again, due to its proximity to the junction, this minor repeat epitope is not present in RTS,S or R21. Thus, our premise is that inducing junctional responses will lead to protective antibodies and can complement repeat antibodies leading to expanded breadth and potency.

Here, we have evaluated the epitopes in the junctional region alone to assess their ability to induce neutralizing antibody responses. These epitopes can be encompassed by short linear peptides [31]; therefore, we have explored strategies to display multiple peptide copies as immunogens. We have previously shown with the HIV-1 envelope fusion peptide that such minimal peptide immunogens, when conjugated to immunogenic carriers, can elicit broad and potent neutralizing responses [35,36,37,38]. In this study we used chikungunya virus-like particles (CHIK-VLP) to display different variations of peptides spanning the PfCSP junctional region. Chikungunya virus (CHIKV) is an enveloped icosahedral alphavirus with quasi T = 4 symmetry and a diameter of 65 nm. The surface E2 protein comprises 240 copies, each with an accessible N-terminus. CHIK-VLP has been shown to be immunogenic in standard test animals [39] and has been demonstrated to be safe and tolerable in a recent human clinical trial [40]. In addition, it has been shown that CHIK-VLP bearing central repeats of PfCSP can elicit protection against malaria challenge [41]. Here, the CHIK-VLP platform was used to evaluate immunogenicity and protection elicited by PfCSP junctional epitope-based immunogen designs.

## 2. Materials and Methods

### 2.1. Epitope Mapping with Overlapping Peptdies

Linear PfCSP repeat epitope maps were created by plotting reciprocal IC_50_ values from peptide competition of mAb binding to recombinant PfCSP, as determined in [34], on the Y-axis and the overlapping peptide number (Table S1) on the X-axis.

### 2.2. Production of CHIK-VLP Immunogens

One day before transfecting Expi293 cells (Thermo Fisher Scientific Inc., Waltham, MA, USA), cells were split with Expi293 serum-free medium (cell density <3 × 10^6^/mL). On the day of transfection, cells were counted and cell density was adjusted to 3 × 10^6^/mL and cell viability >95%. For 400 mL transfections, 340 mL of cells were prepared in a 1 L baffled flask. Plasmid complexes were prepared with ExpiFectamine 293. Specifically: (i) 400 μg plasmid was added in 20 mL Opti-MEM (1×) and filtered, then (ii) 1.08 mL ExpiFectamine 293 was added in 20 mL Opti-MEM. (i) and (ii) were then mixed for about 20 min at room temperature and added to Expi293 cells, cultured in a shaker at 125 rpm/min, 8% CO_2_, and 37 °C. One day after transfection, 2 mL ExpiFectamine enhancer 1 and 20 mL enhancer 2 were added to the cell culture.

### 2.3. Purification of CHIK VLP Immunogens

Four days after transfection, supernatants were collected from transfected cells, centrifuged at 4000 rpm for 30 min at 4 °C, and filtered by using fast pass filter unit. Supernatants (~33 mL) were placed onto a 3 mL OptiPrepTM (Accurate Chemical & Scientific Corporation, Carle Place, NY, USA) at the bottom of an ultracentrifuge tube (Beckman Coulter Inc., Brea, CA, USA), and centrifuged at 20,000 rpm for 2 h at 4 °C. Then, a 6 mL sample of the medium was collected from the bottom, mixed well, and filled into a quick-seal ultracentrifuge tube at 70,000 rpm for 4 h at 4 °C. VLP fractions were collected, filtered, and then purified by using size column Hiprep 26/60 Sephacryl S-500 HR (Merck KGaA, Darmstadt, Germany). VLP peak was collected, concentrated, and filtered, followed by checking OD and running SDS-PAGE.

### 2.4. Production of KLH-p21 Immunogen

KLH displaying peptide 21 was produced by Genscript (GenScript Biotech Corporation, Piscataway, NJ, USA) using sulfydryl conjugation chemistry with a PEG linker as follows: KLH-Thiol-PEG_12_-NPDPNANPNVDPNAN.

### 2.5. Production of Antibody IgGs and Fabs

Antibody expression constructs were synthesized (Gene Universal Inc., Newark, DE, USA) and subcloned into corresponding pVRC8400 vectors. For antibody expression, equal amounts of heavy and light chain plasmid DNA were transfected into Expi293F cells (Thermo Fisher Scientific Inc., Waltham, MA, USA) by using Turbo293 transfection reagent (SPEED Biosystems, Gaithersburg, MD, USA). The transfected cells were cultured in shaker incubator at 120 rpm, 37 °C, and 9% CO_2_ for 5 days. Culture supernatants were harvested and purified over Protein A (GE Healthcare, Chicago, IL, USA) columns. Each antibody was eluted with IgG elution buffer (Thermo Fisher Scientific Inc., Waltham, MA, USA) and immediately neutralized with one tenth volume of 1 M Tris-HCL pH 8.0. The antibodies were then buffer exchanged in PBS by dialysis.

To generate antigen binding fragment (Fab), IgG was incubated with LysC (New England Biolabs, Ipswich, MA, USA) at a ratio of 1 μg LysC per 10 mg IgG at 37 °C overnight. The Fab was purified by collecting flow through from Protein A column followed size-exclusion chromatography.

### 2.6. Antigenic Characterization of Junctional-Epitope–CHIK-VLP Immunogens

A FortéBio Octet HTX instrument (Sartorius, Göttingen, Germany) was used to measure the binding kinetics of conjugates to antibodies. A total of three assay types were used. All CHIK-VLP immunogen assays were performed with phosphate-buffered saline (PBS) with 1% bovine serum albumin (BSA) to minimize nonspecific interactions at 30 °C. Antihuman F_C_ capture (AHC) sensor tips (Sartorius, Göttingen, Germany) were used to capture antibodies for 300 s. Biosensor tips were then equilibrated for 60 s in buffer prior to measuring association with immunogens for 300 s followed by 600 s dissociation. PfCSP was captured using anti-penta his (HIS1K) sensor tips (Sartorius, Göttingen, Germany) for 300 s. Biosensor tips were then equilibrated for 60 s in buffer prior to measuring association with immunogens for 300 s followed by 600 s dissociation. KLH-p21 was characterized using amine-reactive 2nd generation (AR2G) sensor tips (Sartorius, Göttingen, Germany). Biosensor tips were first hydrated for 600 s in nanopure water and then activated using a solution of 20 mM 1-ethyl-3-(3-dimethylamino- propyl) carbodiimide (EDC) and 10 mM sulfo-N-hydroxysuccinimide (S-NHS). KLH-p21 was prepared in 10 mM sodium acetate buffer, pH 4.5, and then immobilized onto the activated biosensor tips for 600 s via the free amino groups. The reaction was quenched using 1 M ethanolamine for 300 s. Prepared tips were then equilibrated for 120 s in kinetics buffer and associated with the antibodies for 300 s followed by 600 s dissociation. Experimental data were analyzed using global fitting with a 1:1 model binding using Octet software, version 9.0 (Sartorius, Göttingen, Germany).

### 2.7. Negative-Stain Electron Microscopy

The sample was diluted to a concentration of approximately 0.05 mg/mL with buffer containing 10 mM HEPES, pH 7.4, and 150 mM NaCl and applied for 15 s to a freshly glow-discharged carbon-coated copper grid. Next, the sample was removed using blotting paper and the grid was washed by applying consecutively three drops of the same buffer and removing them with blotting paper. VLPs that adsorbed to the carbon were negatively stained with 0.75% uranyl acetate. Images were taken using a ThermoFisher Talos F200C transmission electron microscope operated at 200 kV and equipped with a Ceta CCD camera (Thermo Fisher Scientific Inc., Waltham, MA, USA). VLP diameters were measured using e2display.py from the EMAN2 software package [42].

### 2.8. Immunogenicity and Protection Studies

#### 2.8.1. Mice

Female 6- to 8-week old B6(Cg)-Tyrc-2J/J albino mice were obtained from The Jackson Laboratory. All animals were cared for in accordance with American Association for Accreditation of Laboratory Animal Care standards in accredited facilities. All animal procedures were performed according to protocols approved by the Institutional Animal Care and Use Committees of the National Institute of Allergy and Infectious Diseases, National Institutes of Health, specifically: Animal Study Protocol VRC-17-702.

#### 2.8.2. Immunizations

Vaccine immunogens were diluted in sterile PBS to a dose of 5–12 µg with 33.3 µL ALFQ, which, like AS01, comprises a liposomal adjuvant formulation containing monophosphoryl lipid A and QS-21 [43] in a final volume of 50 µL; the KLH-p21 and CHIK-p21_N_ comparison was done with a 25 µg dose. Immunization was performed intramuscularly in the quadriceps at weeks 0 and 3 or 0, 3, and 6, as indicated. Serum sampling was performed 10 days following the 1st immunization and 2 weeks following the 2nd and 3rd immunizations. Challenges were performed 3 weeks following the last immunization, as described below.

#### 2.8.3. Serology

Immune responses were measured to full-length PfCSP or to 15-mer peptides as follows. Immulon 4HBX flat bottom microtiter plates (Thermo Fisher Scientific) were coated with 100 mL per well of full-length PfCSP_SAmut_C5S32 (1.0 mg/mL) in bicarbonate buffer overnight at 4 °C. Coated plates were blocked with 200 mL of PBS + 10% FBS for 2 h at room temperature, followed by incubation for 2 h at 37 °C with 100 mL of sera diluted in blocking buffer (1:20 starting dilution with 10-fold serial dilutions). Plates were washed six times with PBS-Tween20 between each step. For peptide binding, PierceTM streptavidin-coated high-capacity plates (Thermo Fisher Scientific Inc., Waltham, MA, USA) were coated with 100 µL per well of 0.01 µg/mL biotinylated 15-mer peptides (GenScript Biotech Corporation, Piscataway, NJ, USA) diluted in wash buffer (25 mM Tris, 150 mM NaCl, pH 7.2 Tris-buffered saline, 0.1% BSA, and 0.05% Tween-20). Coated plates were incubated for 2 h at 37 °C with 100 µL of sera pooled from each vaccine group, diluted in wash buffer, followed by incubation with 100 µL/well HRP-conjugated goat anti-mouse IgG diluted in wash buffer, as above. Plates were washed three times with 200 µL wash buffer between each step. For peptide and PfCSP ELISAs, plates were incubated with 100 mL/well HRP-conjugated goat anti-mouse IgG (1:6000, Bethyl Laboratories Inc., Montgomery, TX, USA). After a final wash, samples were incubated for 12 min with 1-Step Ultra TMB-ELISA Substrate (Thermo Fisher Scientific Inc., Waltham, MA, USA). The optical density (OD) was read at 450 nm after addition of stopping solution (2N sulfuric acid, 100 mL/well). Mapping using overlapping peptides was performed as previously published [34].

#### 2.8.4. IV Challenge and Quantification of Protection

IV challenges were performed as previously described [34]. Briefly, transgenic *P. berghei* (strain ANKA 676m1c11, MRA-868) expressing full-length *P. falciparum* CSP and a green fluorescent protein/luciferase fusion protein (Pb-PfCSP-GFP/Luc-SPZ) were obtained as previously described [44]. Approximately 3 weeks following the final immunization, mice were intravenously challenged in the tail vein with 2000 freshly harvested Pb-PfCSP-GFP/Luc-SPZ in Leibovitz’s L-15 medium (Thermo Fisher Scientific Inc., Waltham, MA, USA). As a positive control, 300 µg CIS43 IgG was administered via IV 2 h prior to challenge. Then, 40–42 h post-challenge, mice were injected intraperitoneally with 150 μL of D-luciferin (PerkinElmer, Waltham, MA, USA; 30 mg/mL), anesthetized with isoflurane, and imaged with the IVIS^®^ Spectrum in vivo imaging system (PerkinElmer, Waltham, MA, USA) 10 min after luciferin injection. Liver burden was quantified by analyzing a region of interest (ROI) in the upper abdominal region; the total flux (p/s) was measured using the manufacturer’s software (Living Image 4.5, PerkinElmer, Waltham, MA, USA). Percent (%) protection for each mouse was calculated based on the liver burden flux (p/s) data. Percent (%) protection = [100 − ((antibody-treated mouse flux/geometric mean flux of untreated mice) × 100)].

## 3. Results

### 3.1. Design of Immunogens with PfCSP Junctional Residues Displayed on Chikungunya Virus-Like Particles

**Figure 1:**
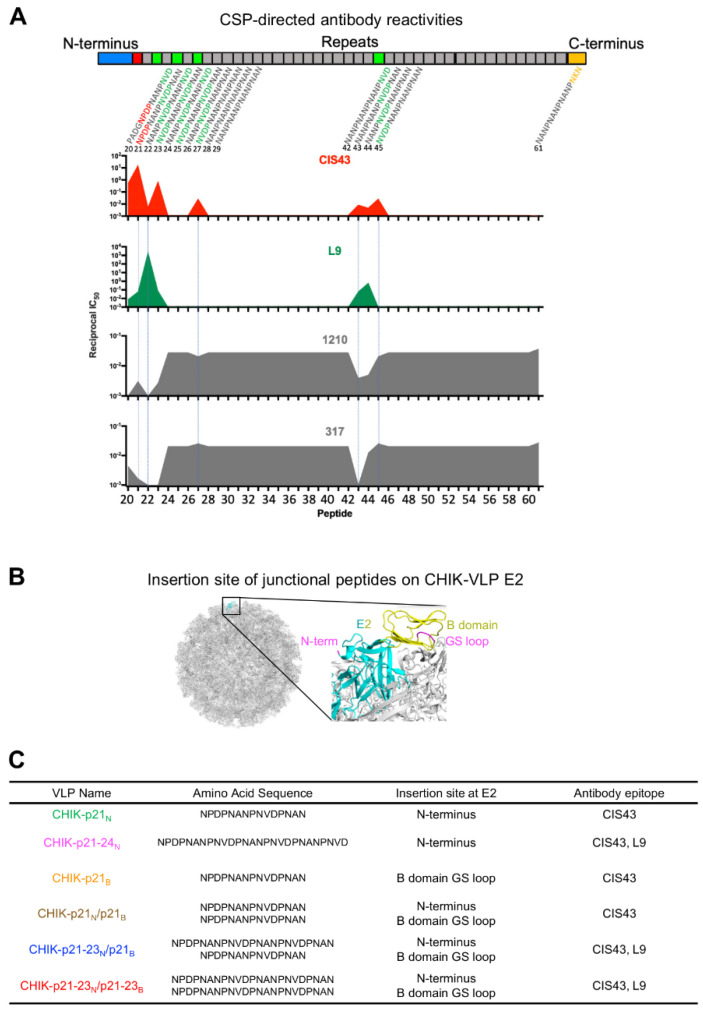
Design of chikungunya virus-like particles (CHIK-VLP) immunogens that incorporate CIS43 and L9 epitopes. (**A**) (Upper panel) Schematic of PfCSP. Tetrapeptide sequences NPDP (red), NVDP (green), and NANP (grey) are highlighted. (Lower panel) Epitope mapping of selected PfCSP repeat-directed mAbs based on competition of their binding to PfCSP by overlapping 15-mer peptides. (**B**) Location of N-terminus and B domain insertion regions on CHIK-VLP E2. (**C**) Summary of CHIK-VLP immunogens incorporating CIS43 and L9 epitopes to N-terminus and/or B domain of CHIK-VLP E2 developed in this study.

The human PfCSP mAbs CIS43 and L9 preferentially target unique junctional epitopes that are found at the interface between the N-terminal and major repeat domains. Short 15-mer overlapping peptides can be used to map the binding of these mAbs, with CIS43 binding to PfCSP being most strongly competed with by peptide 21 (NPDPNANPNVDPNAN) and L9 binding most strongly competed with by peptide 22 (NANPNVDPNANPNVD) (Figure 1A and Table S1, using peptide competition data described in [31]). This is in contrast to mAbs such as 1210 and 317, whose binding is competed by the major repeats (e.g., peptide 29, NANPNANPNANPNAN). Due to the fact that CIS43 and L9 bound strongly to these short peptides, we examined whether they could serve as vaccine minimal immunogens for immunofocusing of PfCSP responses.

We first investigated incorporating variants of the CIS43 epitope into CHIK-VLP, a highly immunogenic nanoparticle [39]. Immunogens were designed with the junctional region peptides displayed on VLPs by genetically fusing PfCSP junctional peptides of different lengths on to the E2 protein of CHIKV, either to the N-terminus and/or to the GS loop in the B domain [45] (Figure 1B,C and Data S1). CIS43 and L9 have 1-2 preferential epitopes in the junction, so we varied the length of the immunogens to see if this would affect immunogenicity. In total, six immunogens were produced, including (1) inserting peptide 21, the primary epitope for CIS43, to the N-terminus of E2 (CHIK-p21_N_); (2) inserting a sequence that spanned from peptide 21 to peptide 24 (p21-24), which contains epitopes for CIS43 and L9, to the N-terminus of E2 (CHIK-p21-24_N_); (3) inserting peptide 21 to the B domain of E2 (CHIK-p21_B_); (4) inserting peptide 21 into the N-terminus and the B domain of E2 (CHIK-p21_N_/p21_B_); (5) inserting a sequence spanning from peptide 21 to peptide 23 (p21-23), which contains epitopes for CIS43 and L9 to the N-terminus of E2, and peptide 21 to the B domain of E2 (CHIK-p21-23_N_/p21_B_); and (6) inserting p21-23 to both the N-terminus and the B domain of E2 (CHIK-p21-23_N_/p21-23_B_).

### 3.2. Electron Microscopic and Antigenic Characterization of CHIK-VLP-Junctional Peptide Immunogens

**Figure 2:**
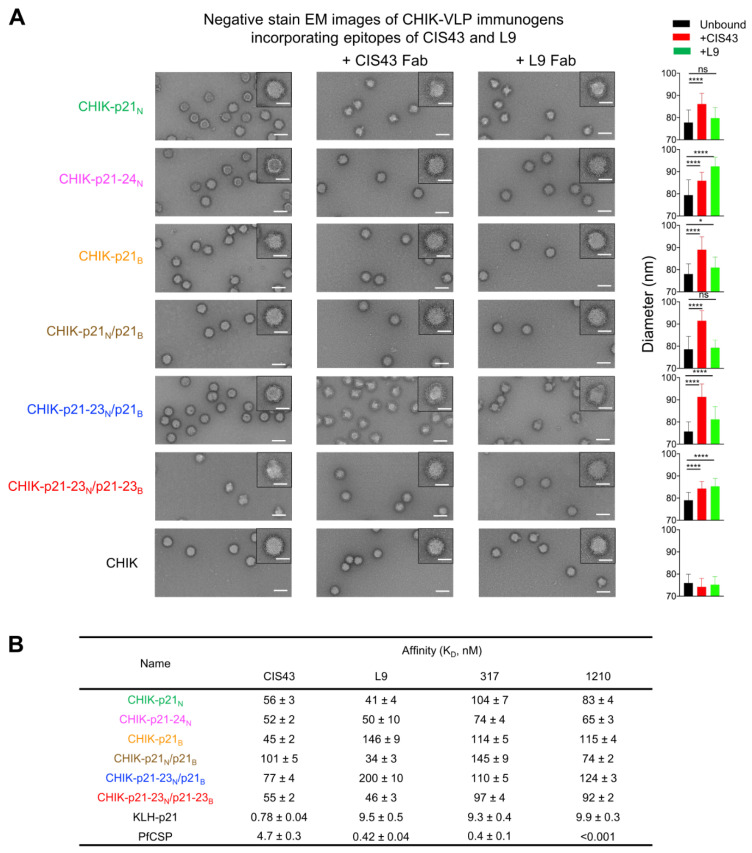
Negative-stain EM and antigenicity of junctional peptide-CHIK-VLP immunogens. (**A**) (Left) Images from negative-stain EM of PfCSP peptide-CHIK-VLP immunogens incorporating CIS43 and L9 epitopes, with and without CIS43 or L9 Fabs. CHIK-VLP without PfCSP peptide (CHIK) was used as control. Scale bars: 100 nm in representative images; 50 nm in insets. (Right) Measured diameters of CHIK-VLP immunogens. Average values are shown in bars with error bar denoting standard deviation. Two-tailed *t*-tests were performed between unbound and CIS43 bound or L9 bound, respectively. *: *p* < 0.05. ****p < 0.0001. ns: *p* ≥ 0.05. (**B**) Affinity of CHIK-VLP immunogens towards antibodies CIS43, L9, 317, and 1210, respectively.

The structural integrity of the VLPs with incorporated PfCSP epitopes was studied using negative-stain electron microscopy (NS-EM). All E2 proteins with inserted PfCSP peptides formed VLPs of expected shape and average diameters in the 75–80 nm range (Figure 2A), indicating that the insertions did not interfere with VLP assembly. NS-EM was then used to detect formation of complexes between antibody CIS43 or L9 and the junctional epitope-bearing VLPs by visualizing mixtures of each VLP with excess of Fab CIS43 or Fab L9 (Figure 2A) and measuring diameters of the particles in the images (Figure 2B). We found that mixing Fab CIS43 with any of the VLPs led to a highly statistically significant increase in particle diameter of at least 5 nm, which was to be expected since each VLP presented the CIS43 epitope. Similar increases were detected when Fab L9 was mixed with VLPs CHIK-p21-24_N_, CHIK-p21-23_N_/p21_B_, and CHIK-p21-23_N_/p21-23_B_, each of which also carried the L9 epitope. In contrast, combining Fab L9 with VLPs CHIK-p21_N_, CHIK-p21_B_, and CHIK-p21_N_/p21_B_, which were devoid of the L9 epitope, did not result in a noticeable increase of particle size, or this increase was below 3 nm. These results indicate that the epitopes of antibodies CIS43 and L9 were presented on the VLPs in accordance with their design and that they were accessible to antibodies on the particle surface and thus, presumably, to B cell receptors.

These CHIK-VLP immunogens were found to bind to mAbs CIS43, L9, 1210, and 317, with sub-micromolar affinities by Bio-Layer Interferometry (BLI), however more promiscuity of binding was observed by this method (Figure 2B and Figure S1). These findings may reflect increased avidity from BLI sensor-immobilized IgG and, in the case of 317, cross-reactivity for the junctional region, which we have previously demonstrated in the context of repeat-truncated PfCSP constructs [34].

### 3.3. Immunogenicity of CHIK-VLP-Junctional Peptide Immunogens

**Figure 3:**
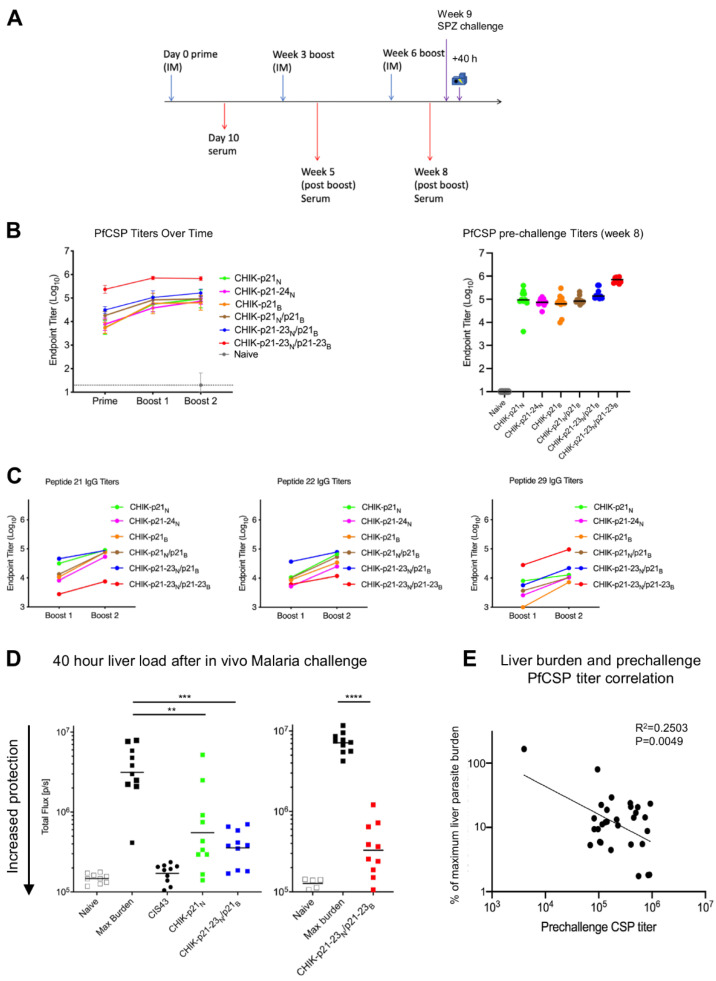
CHIK-VLP immunogens displaying CIS43 and L9 epitopes induced protection against malaria challenge. (**A**) Immunization and challenge schema. Blue arrows denote immunizations and red arrows denote blood draws. Liver burden was quantified 40 h post challenge. (**B**) Left: ELISA titers against PfCSP over time from pooled sera of each group. Right: PfCSP ELISA titers following Boost 2 (pre-challenge time point). (**C**) Serum ELISA titer against peptide 21, peptide 22, and peptide 29. (**D**) Liver burden following malaria challenge with Pb-PfCSP-GFP/Luc-SPZ. CIS43 indicates passively transferred CIS43 IgG, as a positive control. ** *p* < 0.01, *** *p* < 0.001, **** *p* < 0.0001 as calculated by two-tailed Mann–Whitney test. (**E**) Correlation of percent protection from malaria challenge with serum PfCSP ELISA.

The immunogenicity of the CHIK-VLP junctional region immunogens was evaluated by immunizing albino B6 mice with each of the six immunogens, respectively, three times (weeks 0, 3, and 6), and sera was obtained at day 10, week 5, and week 8 (Figure 3A). Among all six immunogens, CHIK-p21-23_N_/p21-23_B_ elicited the highest titer against PfCSP (Figure 3B). After three immunizations, serum responses elicited by most of the CHIK-VLP immunogens showed relatively similar binding to peptide 21 and 22 (containing the preferred epitopes for CIS43 and L9, respectively) but lower responses to the major repeat peptide 29, as expected (Figure 3C). Unexpectedly, CHIK-p21-23_N_/p21-23_B_ elicited the opposite effect, with higher NANP and lower junctional reactivity.

To investigate whether the elicited antibody response could confer protection against malaria challenge, we performed intravenous sporozoite challenges at week 9 for mice immunized with CHIK-p21_N_, CHIK-p21-23_N_/p21_B_, or CHIK-p21-23_N_/p21-23_B_. As a positive control, recombinant CIS43 was infused just prior to challenge and showed a complete reduction in liver burden. In all three immunized groups, the liver load was significantly reduced (Figure 3D), with the group immunized with CHIK-p21-23_N_/p21-23_B_ showing the largest reduction of liver load (~95% reduction). Notably, there was a significant correlation between PfCSP titer and reduction of liver load (*p* = 0.0049, Figure 3E).

To determine if two immunizations were sufficient to confer protection, mice were challenged 3 weeks following two immunizations at weeks 0 and 3. Immunization with CHIK-p21_N_, CHIK-p21-23_N_/p21_B_, or CHIK-p21-23_N_/p21-23_B_ resulted in significant reduction of liver load as compared to the un-immunized group (Figure S2). This protection was similar to that observed after 3 immunizations.

**Figure 4:**
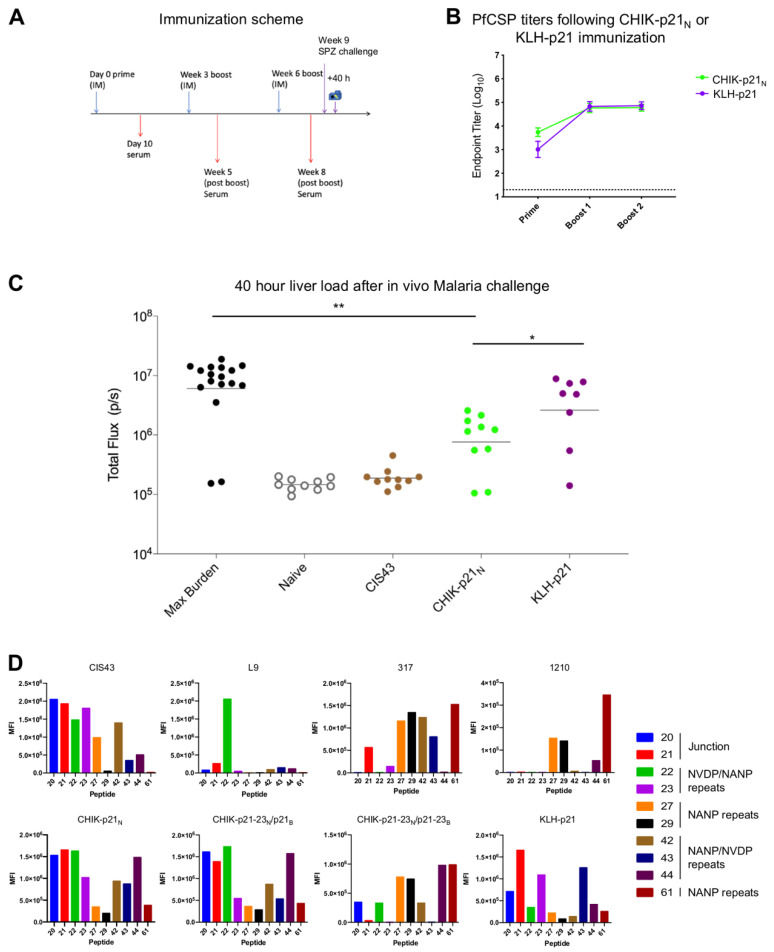
Comparison of malaria protection induced by CHIK-p21 and KLH-p21. (**A**) Immunization and challenge schema. Blue arrows denote immunizations and red arrows denote blood draws. Liver burden was quantified 40 h post challenge. (**B**) Pre-challenge PfCSP ELISA serum titers. (**C**) Liver burden following malaria challenge with Pb-PfCSP-GFP/Luc-SPZ. As a positive control, mAb CIS43 was administered IV prior to challenge. * *p* < 0.05, ** *p* < 0.01 as calculated by two-tailed Mann–Whitney test. (**D**) Mapping to overlapping peptides (described in Table S1) by reference monoclonal antibodies (top row) or vaccine serum post-boost (week 8, bottom row). Week 8 sera from experiments described in Figure 3 was used for CHIK-p21_N_, CHIK-p21-32N/p21_B_, and CHIK-p21-32_N_/p21-23_B_.

Additionally, we compared the effect of displaying the CIS43 minimal immunogen (peptide 21) on the CHIK-VLP to keyhole limpet hemocyanin (KLH) (Figure 4A). Mice immunized with CHIK-p21_N_ and KLH-p21 showed that both vaccines elicited comparable PfCSP binding titers after three immunizations (Figure 4B). However, following sporozoite challenge, the CHIK-p21_N_-immunized group had significantly lower liver load than the KLH-p21-immunzied group (Figure 4C).

Finally, to further investigate the protection elicited by the vaccine constructs, we mapped the serum reactivity using overlapping peptides that spanned the entire central repeat region (Table S1). For reference, representative monoclonal antibodies were mapped to demonstrate a profile of preference for the junction (CIS43), the minor repeats (L9) or the major repeats (317 and 1210) (Figure 4D). As expected, CHIK-p21_N_ and CHIK-p21-23_N_/p21_B_ elicited antibodies that preferentially bound several peptides spanning the junctional region (20–22), and also tolerated the minor repeat sequence, peptide 44. This profile largely matches that of CIS43, as intended. In contrast, KLH-p21 elicited reactivity that was specific to peptide 21. This narrower reactivity profile does not match that of CIS43 as closely, and notably this construct did not provide significant protection. As was initially observed, with reactivity to peptide 21, 22, and 29 (Figure 3C), CHIK-p21-23_N_/p21-23_B_ did not elicit strong binding to the junction or minor repeats, as originally designed. Instead, this construct elicited antibodies that preferentially reacted to peptides containing major repeats, displaying a similar binding profile to 317 and 1210.

## 4. Discussion

In this study, several immunogens were developed based on CHIK-VLPs targeting the junctional region between the PfCSP N-terminus and the major repeat region in an attempt to elicit antibody responses similar to the highly potent human mAbs CIS43 and L9. These immunogens showed well-formed VLPs by electron microscopy and induced strong antibody titers as expected with the ALFQ formulation [46,47], though variable liver burden protection in mice following challenge. Although the junctional and minor repeat regions of PfCSP are known to be sites of neutralization due to mAb characterization [31,32,34], this result is one of the first demonstrations that peptides spanning these epitopes could elicit neutralizing antibodies. The data presented here are in agreement with a recent study by Jelinkova et al., which showed that peptide 21 elicited partial protection when displayed on Qβ bacteriophage VLPs [48]. Similarly, a tetra-branched peptide covering the junctional region has been shown to reduce liver burden following mosquito bite challenge [49]. Here we have further demonstrated the protective effect of VLPs displaying both the CIS43 and L9 epitopes. While no study to date has identified an immunogen that elicits full sterilizing immunity, even partial protection following the relatively strenuous challenge of intravenous sporozoites used here validates the concept of targeting these epitopes within PfCSP.

Of all the immunogens tested, CHIK-p21-23_N_/p21-23_B_ tended to be the most promising, both in the magnitude of its immunogenicity and its ability to reduce liver parasite load following challenge. Interestingly, CHIK-p21-23_N_/p21-23_B_ elicited higher titers against the NANP major repeats and lower responses to the junctional and minor repeat epitopes, more closely resembling mAbs like 317, which are cross-reactive but have preferred binding to the major repeats. These data lend credence to the idea that eliciting cross-reactivity across the various epitopes in the repeat region may be the best strategy to induce protection [48,50]. Interestingly, CHIK-p21-23_N_/p21-23_B_ does not display the “NPNA” structural motif bound by mAbs targeting the major repeats [26,51]. In contrast, very similar constructs, such as CHIK-p21-23_N_/p21_B_ (a shorter peptide inserted in the E2 B domain being the only difference), elicited preferential reactivity to the junctional region, as designed. It seems likely, therefore, that the structural idiosyncrasies of each construct result in the differential scaffolding of the “NPN” motif, which is the structural core often recognized by PfCSP repeat mAbs [31,32,33]. Further study is required to understand the underlying mechanisms of protection from CHIK-p21_N_ compared to CHIK-p21-23_N_/p21-23_B_, specifically the degree to which protection derives from reactivity to the junctional or major repeat epitopes.

The immunogens developed here provide the initial basis for further development of CHIK or other VLP-expressing systems for displaying the epitopes of the potent junctional- and minor repeat-targeting mAbs, CIS43 and L9, respectively. As the CHIK-VLP immunogens developed here displayed PfCSP regions distinct from the RTS,S and R21 immunogens (displaying the major repeat and C-terminal domains), they could be potentially be co-administered to increase the epitope breadth of responses against PfCSP [8]. Alternatively, the immunogen sequence from R21 or other promising PfCSP-based vaccines, such as the tobacco mosaic virus platform [52], could be redesigned to incorporate the junctional regions described here, which could expand the breadth of responses. Finally, heterologous prime-boost regimens focusing first on junctional epitopes, then on major or minor repeat regions, may further mature cross-reactivity that has been associated with PfCSP-neutralizing responses [26,31,34,50].

Taken together, the data presented here compel further design of PfCSP immunogens with the goal of improving the breadth of responses to PfCSP, compared to the benchmark of RTS,S. Given that PfCSP is the only known target for pre-erythrocytic malaria vaccination, and the need to improve upon the potency of RTS,S, the incorporation of additional neutralizing epitopes as described here may be key to a second-generation malaria PfCSP vaccine.

## Data Availability

The data presented in this study are available in this article or its supplementary materials.

## Supplementary Materials

The following are available online at https://www.mdpi.com/2076-393X/9/3/272/s1: Figure S1: Binding curves and fit for KD measurements of CHIK-VLP immunogens towards antibodies CIS43, L9, 317, and 1210, respectively, using BLI, Figure S2: Malaria protection following two immunizations of CHIK-VLP immunogens displaying, Table S1: Overlapping 15-mer peptides to the repeat region of PfCSP, strain 3D7, Data S1: Amino acid sequences of six junctional epitope-CHIK VLP immunogens.
