# Supplementary material for: Design of Alphavirus Virus-Like Particles Presenting Circumsporozoite Junctional Epitopes That Elicit Protection against Malaria
Source: Vaccines (Basel). 2021 Mar 18;9(3):272. doi: 10.3390/vaccines9030272 (PMC8003078; doi:10.3390/vaccines9030272)
Supplement: Supplementary file 1 [file vaccines-09-00272-s001.pdf]

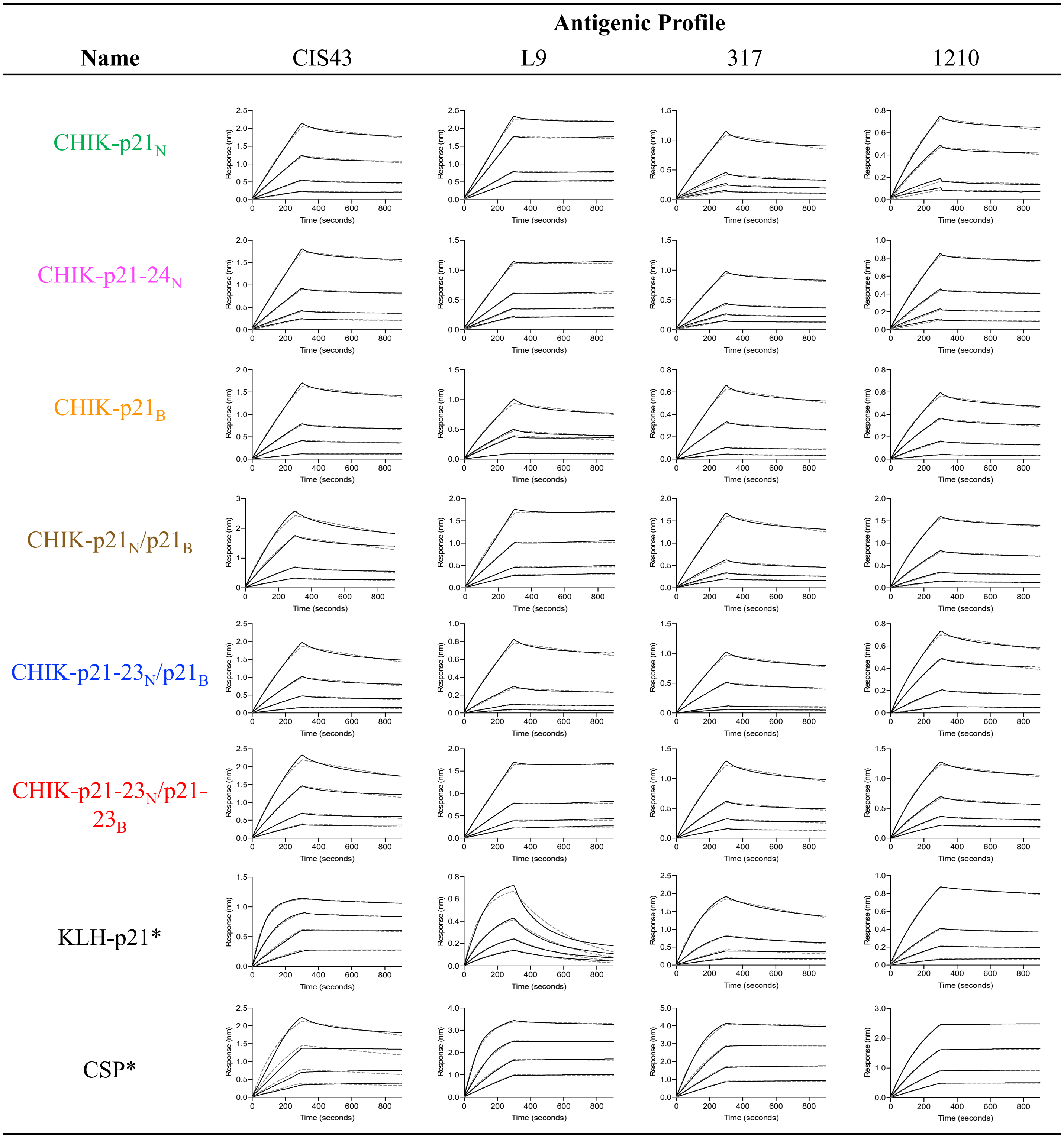

Figure S1: Binding curves and fit for KD measurements of CHIK-VLP immunogens towards antibodies CIS43, L9, 317, and 1210, respectively, using BLI. Biolayer interferometry (BLI) curves for CHIK-VLP immunogens towards antibodies CIS43, L9, 317, and 1210, respectively. Solid lines are the real processed data, and the dashed lines are the curve fits that were used to obtain the K_D values. Curves were modeled using 1:1 binding stoichiometry and the resulting kinetics were used to obtain K_D values. Dilution series started at 500 nM with 2-fold dilutions. *: KLH-p21 and CSP could not be fit to the 1:1 binding model used for the other immunogens. To circumvent this issue, KLH-p21 was captured onto amine-reactive biosensors, while CSP was immobilized via the 6xHis tag. Each immunogen was then dipped into a solution of the antibody.

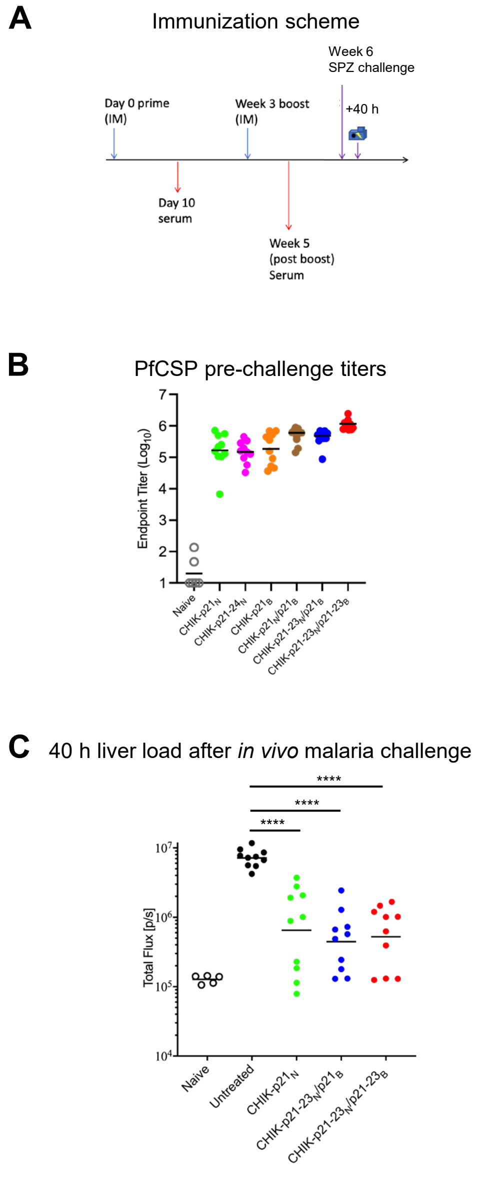

Figure S2. Malaria protection following two immunizations of CHIK-VLP immunogens displaying CIS43 and L9 epitopes. (A) Immunization and challenge schema. Blue arrows denote immunizations and red arrows denote blood draws. Liver burden was quantified 40 hours post challenge. **(B)** Pre-challenge PfCSP ELISA serum titers. **(C)** Liver burden following malaria challenge with Pb-PfCSP-GFP/Luc-SPZ. **** $P<0.0001$ as calculated by two tailed Mann-Whitney test.

Peptide number	Peptide sequence
20	^{97}PADGNPDPNANPNVD^{111}
21	^{101}NPDPNANPNVDPNAN^{115}
22	^{105}NANPNVDPNANPNVD^{119}
23	^{109}NVDPNANPNVDPNAN^{123}
24	^{113}NANPNVDPNANPNVD^{127}
25	^{117}NVDPNANPNVDPNAN^{131}
26	^{121}NANPNVDPNANPNAN^{135}
27	^{125}NVDPNANPNANPNAN^{139}
28	^{129}NANPNANPNANPNAN^{143}
29-41	NANPNANPNANPNAN
42	^{185}NANPNANPNANPNVD^{199}
43	^{189}NANPNANPNVDPNAN^{203}
44	^{193}NANPNVDPNANPNAN^{207}
45	^{197}NVDPNANPNANPNAN^{211}
46-60	NANPNANPNANPNAN
61	^{261}NANPNANPNANPNKN^{275}

Table S1. Overlapping 15-mer peptides to the repeat region of PfCSP, strain 3D7. Superscripts indicate N-terminal amino acid numbering. Note that the following peptides are identical: 22 and 24; 23 and 25; 26 and 44; 27 and 45; 28-41, 46-60.

Data S1. Amino acid sequences of six junctional epitope-CHIK VLP immunogens.
Junctional epitope residues are highlighted in red.

CHIK-p21$_{N}$

MEFIPTQTFYNRRYQPRPWAPRPTIQVIRPRPRPQRQAGQLAQLISAVNKLTMRAVPQQKPRRN
RKNKKQRQKKQAPQNDPKQKKQPPQKKPAQKKKKPGRRERMCMKIENDCIFEVKHEGKVMGYAC
LVGDKVMKPAHVKGTIDNADLAKLAFKRSSKYDLECAQIPVHMKSDASKFTHEKPEGYYNWHHG
AVQYSGGRFTIPTGAGKPGDSGRPIFDNKGRVVAIVLGGANEGARTALSVVTWNKDIVTKITPE
GAEEWSLALPVLCLLANTTFPCSQPPCTPCCYEKEPESTLRMLEDNVMRPGYYQLLKASLTCSP
HRQRR**NPDPNANPNVDPNAN**STKDNFNVYKATRPYLAHCPDCGEGHSCHSPIALERIRNEATDG
TLKIQVSLQIGIKTDDSHDWTKLRYMDSHTPADAERAGLLVRTSAPCTITGTMGHFILARCPKG
ETLTVGFTDSRKISHTCTHPFHHEPPVIGRERFHSRPQHGKELPCSTYVQSTAATAEEIEVHMP
PDTPDRTLMTQQSGNVKITVNGQTVRYKCNCGGSSGSGSNEGLTTTDKVINNCKIDQCHAAVTN
HKNWQYNSPLVPRNAELGDRKGKIHIPFPLANVTCRVPKARNPTVTYGKNQVTMLLYPDHPTLL
SYRNMGQEPNYHEEWVTHKKEVTLTVPTEGLEVTWGNNEPYKYWPQMSTNGTAHGHPHEIILYY
YELYPTMTVVIVSVASFVLLSMVGTAVGMCVCARRRCITPYELTPGATVPFLLSLLCCVRTTKA
ATYYEAAAYLWNEQQPLFWLQALIPLAALIVLCNCLKLLPCCCKTLAFLAVMSIGAHTVSAYEH
VTVIPNTVGVPYKTLVNRPGYSPMVLEMELQSVTLEPTLSLDYITCEYKTVIPSPYVKCCGTAE
CKDKSLPDYSCKVFTGVYPFMWGGAYCFCDAENTQLSEAHVEKSESCKTEFASAYRAHTASASA
KLRVLYQGNNITVAAYANGDHAVTVKDAKFVVGPMSSAWTPFDNKIVVYKGDVYNMDYPPFGAG
RPGQFGDIQSRTPESKDVYANTQLVLQRPAAGTVHVPYSQAPSGFKYWLKERGASLQHTAPFGC
QIATNPVRAVNCAVGNIPISIDIPDAAFTRVVDAPSVTDMSCEVPACTHSSDFGGVAIIKYTAS
KKGKCAVHSMTNAVTIREADVEVEGNSQLQISFSTALASAEFRVQVCSTQVHCAAACHPPKDHI
VNYPASHTTLGVQDISTTAMSWVQKITGGVGLIVAVAALILIVVLCVSFSRH

CHIK-p21-24$_{N}$

MEFIPTQTFYNRRYQPRPWAPRPTIQVIRPRPRPQRQAGQLAQLISAVNKLTMRAVPQQKPRRN
RKNKKQRQKKQAPQNDPKQKKQPPQKKPAQKKKKPGRRERMCMKIENDCIFEVKHEGKVMGYAC
LVGDKVMKPAHVKGTIDNADLAKLAFKRSSKYDLECAQIPVHMKSDASKFTHEKPEGYYNWHHG
AVQYSGGRFTIPTGAGKPGDSGRPIFDNKGRVVAIVLGGANEGARTALSVVTWNKDIVTKITPE
GAEEWSLALPVLCLLANTTFPCSQPPCTPCCYEKEPESTLRMLEDNVMRPGYYQLLKASLTCSP
HRQRR**NPDPNANPNVDPNANPNVDPNANPNVDPNAN**STKDNFNVYKATRPYLAHCPDCGEGHSC
HSPIALERIRNEATDGTLKIQVSLQIGIKTDDSHDWTKLRYMDSHTPADAERAGLLVRTSAPCT
ITGTMGHFILARCPKGETLTVGFTDSRKISHTCTHPFHHEPPVIGRERFHSRPQHGKELPCSTY
VQSTAATAEEIEVHMPPDTPDRTLMTQQSGNVKITVNGQTVRYKCNCGGSSGSGSNEGLTTTDK
VINNCKIDQCHAAVTNHKNWQYNSPLVPRNAELGDRKGKIHIPFPLANVTCRVPKARNPTVTYG
KNQVTMLLYPDHPTLLSYRNMGQEPNYHEEWVTHKKEVTLTVPTEGLEVTWGNNEPYKYWPQMS
TNGTAHGHPHEIILYYYELYPTMTVVIVSVASFVLLSMVGTAVGMCVCARRRCITPYELTPGAT
VPFLLSLLCCVRTTKAATYYEAAAYLWNEQQPLFWLQALIPLAALIVLCNCLKLLPCCCKTLAF
LAVMSIGAHTVSAYEHVTVIPNTVGVPYKTLVNRPGYSPMVLEMELQSVTLEPTLSLDYITCEY

KTVIPSPYVKCCGTAECKDKSLPDYSCKVFTGVYPFMWGGAYCFCDAENTQLSEAHVEKSESCK
TEFASAYRAHTASASAKLRVLYQGNNITVAAYANGDHAVTVKDAKFVVGPMSSAWTPFDNKIVV
YKGDVYNMDYPPFGAGRPGQFGDIQSRTPESKDVYANTQLVLQRPAAGTVHVPYSQAPSGFKYW
LKERGASLQHTAPFGCQIATNPVRAVNCAVGNIPISIDIPDAAFTRVVDAPSVTDMSCEVPACT
HSSDFGGVAIIKYTASKKGKCAVHSMTNAVTIREADVEVEGNSQLQISFSTALASAEFRVQVCS
TQVHCAAACHPPKDHIVNYPASHTTLGVQDISTTAMSWVQKITGGVGLIVAVAALILIVVLCVS
FSRH

CHIK-p21$_{B}$

MEFIPTQTFYNRRYQPRPWAPRPTIQVIRPRPRPQRQAGQLAQLISAVNKLTMRAVPQQKPRRN
RKNKKQRQKKQAPQNDPKQKKQPPQKKPAQKKKKPGRRERMCMKIENDCIFEVKHEGKVMGYAC
LVGDKVMKPAHVKGTIDNADLAKLAFKRSSKYDLECAQIPVHMKSDASKFTHEKPEGYYNWHHG
AVQYSGGRFTIPTGAGKPGDSGRPIFDNKGRVVAIVLGGANEGARTALSVVTWNKDIVTKITPE
GAEEWSLALPVLCLLANTTFPCSQPPCTPCCYEKEPESTLRMLEDNVMRPGYYQLLKASLTCSP
HRQRRSTKDNFNVYKATRPYLAHCPDCGEGHSCHSPIALERIRNEATDGTLKIQVSLQIGIKTD
DSHDWTKLRYMDSHTPADAERAGLLVRTSAPCTITGTMGHFILARCPKGETLTVGFTDSRKISH
TCTHPFHHEPPVIGRERFHSRPQHGKELPCSTYVQSTAATAEEIEVHMPPDTPDRTLMTQQSGN
VKITVNGQTVRYKCNCGGS**NPDPNANPNVDPNAN**SGSGSNEGLTTTDKVINNCKIDQCHAAVTN
HKNWQYNSPLVPRNAELGDRKGKIHIPFPLANVTCRVPKARNPTVTYGKNQVTMLLYPDHPTLL
SYRNMGQEPNYHEEWVTHKKEVTLTVPTEGLEVTWGNNEPYKYWPQMSTNGTAHGHPHEIILYY
YELYPTMTVVIVSVASFVLLSMVGTAVGMCVCARRCITPYELTPGATVPFLLSLLCCVRTTKA
ATYYEAAAYLWNEQQPLFWLQALIPLAALIVLCNCLKLLPCCCKTLAFLAVMSIGAHTVSAYEH
VTVIPNTVGVPYKTLVNRPGYSPMVLEMELQSVTLEPTLSLDYITCEYKTVIPSPYVKCCGTAE
CKDKSLPDYSCKVFTGVYPFMWGGAYCFCDAENTQLSEAHVEKSESCKTEFASAYRAHTASASA
KLRVLYQGNNITVAAYANGDHAVTVKDAKFVVGPMSSAWTPFDNKIVVYKGDVYNMDYPPFGAG
RPGQFGDIQSRTPESKDVYANTQLVLQRPAAGTVHVPYSQAPSGFKYWLKERGASLQHTAPFGC
QIATNPVRAVNCAVGNIPISIDIPDAAFTRVVDAPSVTDMSCEVPACTHSSDFGGVAIIKYTAS
KKGKCAVHSMTNAVTIREADVEVEGNSQLQISFSTALASAEFRVQVCSTQVHCAAACHPPKDHI
VNYPASHTTLGVQDISTTAMSWVQKITGGVGLIVAVAALILIVVLCVSFSRH

CHIK-p21$_{N}$/p21$_{B}$

MEFIPTQTFYNRRYQPRPWAPRPTIQVIRPRPRPQRQAGQLAQLISAVNKLTMRAVPQQKPRRN
RKNKKQRQKKQAPQNDPKQKKQPPQKKPAQKKKKPGRRERMCMKIENDCIFEVKHEGKVMGYAC
LVGDKVMKPAHVKGTIDNADLAKLAFKRSSKYDLECAQIPVHMKSDASKFTHEKPEGYYNWHHG
AVQYSGGRFTIPTGAGKPGDSGRPIFDNKGRVVAIVLGGANEGARTALSVVTWNKDIVTKITPE
GAEEWSLALPVLCLLANTTFPCSQPPCTPCCYEKEPESTLRMLEDNVMRPGYYQLLKASLTCSP
HRQRR**NPDPNANPNVDPNAN**STKDNFNVYKATRPYLAHCPDCGEGHSCHSPIALERIRNEATDG
TLKIQVSLQIGIKTDDSHDWTKLRYMDSHTPADAERAGLLVRTSAPCTITGTMGHFILARCPKG
ETLTVGFTDSRKISHTCTHPFHHEPPVIGRERFHSRPQHGKELPCSTYVQSTAATAEEIEVHMP
PDTPDRTLMTQQSGNVKITVNGQTVRYKCNCGGS**NPDPNANPNVDPNAN**SGSGSNEGLTTTDKV

INNCKIDQCHAAVTNHKNWQYNSPLVPRNAELGDRKGKIHIPFPLANVTCRVPKARNPTVTYGK
NQVTMLLYPDHPTLLSYRNMGQEPNYHEEWVTHKKEVTLTVPTEGLEVTWGNNEPYKYWPQMST
NGTAHGHPHEIILYYYELYPTMTVVIVSVASFVLLSMVGTAVGMCVCARRRCITPYELTPGATV
PFLLSLLCCVRTTKAATYYEAAAYLWNEQQPLFWLQALIPLAALIVLCNCLKLLPCCCKTLAFL
AVMSIGAHTVSAYEHVTVIPNTVGVPYKTLVNRPGYSPMVLEMELQSVTLEPTLSLDYITCEYK
TVIPSPYVKCCGTAECKDKSLPDYSCKVFTGVYPFMWGGAYCFCDAENTQLSEAHVEKSESCKT
EFASAYRAHTASASAKLRVLYQGNNITVAAYANGDHAVTVKDAKFVVGPMSSAWTPFDNKIVVY
KGDVYNMDYPPFGAGRPGQFGDIQSRTPESKDVYANTQLVLQRPAAGTVHVPYSQAPSGFKYWL
KERGASLQHTAPFGCQIATNPVRAVNCAVGNIPISIDIPDAAFTRVVDAPSVTDMSCEVPACTH
SSDFGGVAIIKYTASKKGKCAVHSMTNAVTIREADVEVEGNSQLQISFSTALASAEFRVQVCST
QVHCAAACHPPKDHIVNYPASHTTLGVQDISTTAMSWVQKITGGVGLIVAVAALILIVVLCVSF
SRH

CHIK-p21-23$_N$/p21$_B$

MEFIPTQTFYNRRYQPRPWAPRPTIQVIRPRPRPQRQAGQLAQLISAVNKLTMRAVPQQKPRRN
RKNKKQRQKKQAPQNDPKQKKQPPQKKPAQKKKKPGRRERMCMKIENDCIFEVKHEGKVMGYAC
LVGDKVMKPAHVKGTIDNADLAKLAFKRSSKYDLECAQIPVHMKSDASKFTHEKPEGYYNWHHG
AVQYSGGRFTIPTGAGKPGDSGRPIFDNKGRVVAIVLGGANEGARTALSVVTWNKDIVTKITPE
GAEEWSLALPVLCLLANTTFPCSQPPCTPCCYEKEPESTLRMLEDNVMRPGYYQLLKASLTCSP
HRQRR**NPDPNANPNVDPNANPNVDPNAN**STKDNFNVYKATRPYLAHCPDCGEGHSCHSPIALER
IRNEATDGTLKIQVSLQIGIKTDDSHDWTKLRYMDSHTPADAERAGLLVRTSAPCTITGTMGHF
ILARCPKGETLTVGFTDSRKISHTCTHPFHHEPPVIGRERFHSRPQHGKELPCSTYVQSTAATA
EEIEVHMPPDTPDRTLMTQQSGNVKITVNGQTVRYKCNCGGS**NPDPNANPNVDPNAN**SGSGSNE
GLTTTDKVINNCKIDQCHAAVTNHKNWQYNSPLVPRNAELGDRKGKIHIPFPLANVTCRVPKAR
NPTVTYGKNQVTMLLYPDHPTLLSYRNMGQEPNYHEEWVTHKKEVTLTVPTEGLEVTWGNNEPY
KYWPQMSTNGTAHGHPHEIILYYYELYPTMTVVIVSVASFVLLSMVGTAVGMCVCARRRCITPY
ELTPGATVPFLLSLLCCVRTTKAATYYEAAAYLWNEQQPLFWLQALIPLAALIVLCNCLKLLPC
CCKTLAFLAVMSIGAHTVSAYEHVTVIPNTVGVPYKTLVNRPGYSPMVLEMELQSVTLEPTLSL
DYITCEYKTVIPSPYVKCCGTAECKDKSLPDYSCKVFTGVYPFMWGGAYCFCDAENTQLSEAHV
EKSESCKTEFASAYRAHTASASAKLRVLYQGNNITVAAYANGDHAVTVKDAKFVVGPMSSAWTP
FDNKIVVYKGDVYNMDYPPFGAGRPGQFGDIQSRTPESKDVYANTQLVLQRPAAGTVHVPYSQA
PSGFKYWLKERGASLQHTAPFGCQIATNPVRAVNCAVGNIPISIDIPDAAFTRVVDAPSVTDMS
CEVPACTHSSDFGGVAIIKYTASKKGKCAVHSMTNAVTIREADVEVEGNSQLQISFSTALASAE
FRVQVCSTQVHCAAACHPPKDHIVNYPASHTTLGVQDISTTAMSWVQKITGGVGLIVAVAALIL
IVVLCVSFSRH

CHIK-p21-23$_N$/p21-23$_B$

MEFIPTQTFYNRRYQPRPWAPRPTIQVIRPRPRPQRQAGQLAQLISAVNKLTMRAVPQQKPRRN
RKNKKQRQKKQAPQNDPKQKKQPPQKKPAQKKKKPGRRERMCMKIENDCIFEVKHEGKVMGYAC
LVGDKVMKPAHVKGTIDNADLAKLAFKRSSKYDLECAQIPVHMKSDASKFTHEKPEGYYNWHHG

AVQYSGGRFTIPTGAGKPGDSGRPIFDNKGRVVAIVLGGANEGARTALSVVTWNKDIVTKITPE
GAEEWSLALPVLCLLANTTFPCSQPPCTPCCYEKEPESTLRMLEDNVMRPGYYQLLKASLTCSP
HRQRR**NPDPNANPNVDPNANPNVDPNAN**STKDNFNVYKATRPYLAHCPDCGEGHSCHSPIALER
IRNEATDGTLKIQVSLQIGIKTDDSHDWTKLRYMDSHTPADAERAGLLVRTSAPCTITGTMGHF
ILARCPKGETLTVGFTDSRKISHTCTHPFHHEPPVIGRERFHSRPQHGKELPCSTYVQSTAATA
EEIEVHMPPDTPDRTLMTQQSGNVKITVNGQTVRYKCNCGGS**NPDPNANPNVDPNANPNVDPNA**
NSGSGSNEGLTTTDKVINNCKIDQCHAAVTNHKNWQYNSPLVPRNAELGDRKGKIHIPFPLANV
TCRVPKARNPTVTYGKNQVTMLLYPDHPTLLSYRNMGQEPNYHEEWVTHKKEVTLTVPTEGLEV
TWGNNEPYKYWPQMSTNGTAHGHPHEIILYYYELYPTMTVVIVSVASFVLLSMVGTAVGMCVCA
RRRCITPYELTPGATVPFLLSLLCCVRTTKAATYYEAAAYLWNEQQPLFWLQALIPLAALIVLC
NCLKLLPCCCKTLAFLAVMSIGAHTVSAYEHVTVIPNTVGVPYKTLVNRPGYSPMVLEMELQSV
TLEPTLSLDYITCEYKTVIPSPYVKCCGTAECKDKSLPDYSCKVFTGVYPFMWGGAYCFCDAEN
TQLSEAHVEKSESCKTEFASAYRAHTASASAKLRVLYQGNNITVAAYANGDHAVTVKDAKFVVG
PMSSAWTPFDNKIVVYKGDVYNMDYPPFGAGRPGQFGDIQSRTPESKDVYANTQLVLQRPAAGT
VHVPYSQAPSGFKYWLKERGASLQHTAPFGCQIATNPVRAVNCAVGNIPISIDIPDAAFTRVVD
APSVTDMSCEVPACTHSSDFGGVAIIKYTASKKGKCAVHSMTNAVTIREADVEVEGNSQLQISF
STALASAEFRVQVCSTQVHCAAACHPPKDHIVNYPASHTTLGVQDISTTAMSWVQKITGGVGLI
VAVAALILIVVLCVSFSRH
